# Dietary nitrate and population health: a narrative review of the translational potential of existing laboratory studies

**DOI:** 10.1186/s13102-021-00292-2

**Published:** 2021-06-07

**Authors:** Oliver M. Shannon, Chris Easton, Anthony I. Shepherd, Mario Siervo, Stephen J. Bailey, Tom Clifford

**Affiliations:** 1 Human Nutrition Research Centre, Population Health Sciences Institute, Newcastle University, Newcastle upon Tyne, UK; 2 Institute for Clinical Exercise and Health Science, University of the West of Scotland, Blantyre, Scotland, UK; 3 School of Sport, Health & Exercise Science, University of Portsmouth, Portsmouth, UK; 4 School of Life Sciences, The University of Nottingham Medical School, Queen’s Medical Centre, Nottingham, UK; 5 School of Sport, Exercise and Health Sciences, Loughborough University, Loughborough, UK

**Keywords:** Nitrate, Beetroot juice, Population health, Epidemiology, Randomised controlled trials, Blood pressure, Exercise performance, Translation

## Abstract

**Background:**

Dietary inorganic nitrate (NO_3_^−^) is a polyatomic ion, which is present in large quantities in green leafy vegetables and beetroot, and has attracted considerable attention in recent years as a potential health-promoting dietary compound. Numerous small, well-controlled laboratory studies have reported beneficial health effects of inorganic NO_3_^−^ consumption on blood pressure, endothelial function, cerebrovascular blood flow, cognitive function, and exercise performance. Translating the findings from small laboratory studies into ‘real-world’ applications requires careful consideration.

**Main body:**

This article provides a brief overview of the existing empirical evidence basis for the purported health-promoting effects of dietary NO_3_^−^ consumption. Key areas for future research are then proposed to evaluate whether promising findings observed in small animal and human laboratory studies can effectively translate into clinically relevant improvements in population health. These proposals include: 1) conducting large-scale, longer duration trials with hard clinical endpoints (e.g. cardiovascular disease incidence); 2) exploring the feasibility and acceptability of different strategies to facilitate a prolonged increase in dietary NO_3_^−^ intake; 3) exploitation of existing cohort studies to explore associations between NO_3_^−^ intake and health outcomes, a research approach allowing larger samples sizes and longer duration follow up than is feasible in randomised controlled trials; 4) identifying factors which might account for individual differences in the response to inorganic NO_3_^−^ (e.g. sex, genetics, habitual diet) and could assist with targeted/personalised nutritional interventions; 5) exploring the influence of oral health and medication on the therapeutic potential of NO_3_^−^ supplementation; and 6) examining potential risk of adverse events with long term high- NO_3_^−^ diets.

**Conclusion:**

The salutary effects of dietary NO_3_^−^ are well established in small, well-controlled laboratory studies. Much less is known about the feasibility and efficacy of long-term dietary NO_3_^−^ enrichment for promoting health, and the factors which might explain the variable responsiveness to dietary NO_3_^−^ supplementation between individuals. Future research focussing on the translation of laboratory data will provide valuable insight into the potential applications of dietary NO_3_^−^ supplementation to improve population health.

## Background

**Fig. 1:**
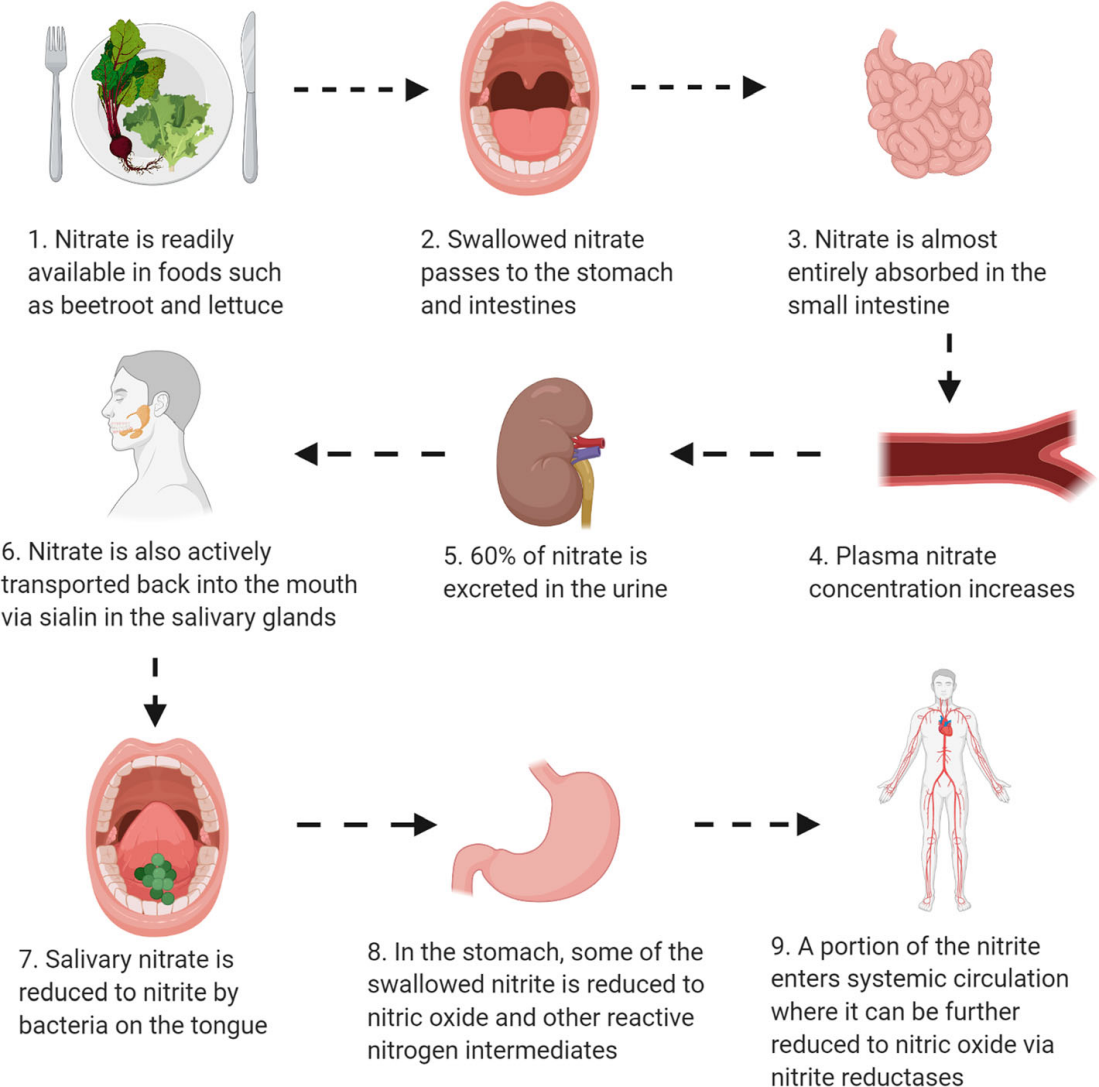
A schematic representation of the nitrate-nitrite-nitric oxide pathway. Created with Biorender.com

Dietary inorganic nitrate (NO_3_^−^) is a polyatomic ion present in large quantities in green leafy vegetables and certain root vegetables such as beetroot [1]. In recent years, inorganic NO_3_^−^has attracted substantial attention as a potential health promoting and exercise performance-enhancing dietary compound. These effects have largely been attributed to its ability to serve as a substrate for the ubiquitous gasotransmitter, nitric oxide (NO; Fig. 1) [2]. Following consumption, inorganic NO_3_^−^is absorbed in the upper gastrointestinal tract, increasing plasma NO_3_^−^ concentration [3]. In the blood, exogenous NO_3_^−^ mixes with endogenous NO_3_^−^ produced via oxidation of NO. Most (~ 60%) of the ingested NO_3_^−^ is excreted in the urine [4]. However, ~ 25% is actively taken up by the salivary glands via the transporter protein sialin [5], and secreted into the oral cavity, where it is reduced to nitrite (NO_2_^−^) by facultative anaerobic bacteria residing primarily on the dorsal surface of the tongue [6, 7]. Salivary (in the saliva) NO_2_^−^ is then swallowed and a portion is converted into NO and other nitrogen oxides in the acidic environment of the stomach [2, 8, 9]. A further portion of the swallowed NO_2_^−^ reaches the systemic circulation, where it can be transported to various tissues and reduced to NO by a range of enzymatic and non-enzymatic catalysis [2, 3]. By increasing the bioavailability of NO and other nitrogen oxides, which play a role in the regulation of multifarious physiological processes, inorganic NO_3_^−^ has the capacity to elicit far-reaching effects in the human body.

One of the most well-documented effects following inorganic NO_3_^−^ consumption is a decrease in blood pressure (BP), an effect which was first demonstrated by Larsen and colleagues from the Karolinska Institute in 2006 [10]. This group reported that 3 days of supplementation with NO_3_^−^ salts (0.1 mmol/kg/d sodium NO_3_^−^) reduced diastolic and mean arterial BP by − 3.7 and − 3.2 mmHg, respectively, in young healthy adults. A number of independent research groups [11–16] has substantiated these promising findings across a range of participant cohorts and using various supplementation strategies, including the provision of whole and juiced vegetables, especially beetroot juice [17]. Over the past 10 years, as this burgeoning research area has expanded, various other potentially beneficial effects of inorganic NO_3_^−^ consumption have also emerged. Notably, NO_3_^−^ has been shown to improve a range of cardiovascular risk factors [17], increasing endothelial function [14, 18–21], decreasing arterial stiffness [15, 20, 22, 23], and reducing platelet aggregation [20, 24, 25]. Some [26–28], but not all [18, 29–31] studies have also shown beneficial effects of inorganic NO_3_^−^ on cognitive function – effects which may be underpinned by alterations in cerebrovascular blood flow [31–33] and could be of value to a range of clinical and healthy populations [34]. Likewise, NO_3_^−^ has been identified as a potential prebiotic for the oral microbiome [35], with the potential to positively impact oral health [36]. Moreover, NO_3_^−^ consumption has been demonstrated to improve performance during continuous [12, 13, 29, 37–42], intermittent [43–45] and strength-based [46, 47] exercise, especially in untrained and recreationally active individuals [40, 48–50]. The mechanisms for the ergogenic effects of NO_3_^−^ have not been fully resolved, but may include: 1) improvements in mitochondrial efficiency (reported by some [51], but not others [52]); 2) enhanced muscle contractile efficiency/ function [53–56]; and 3) augmented tissue blood flow, particularly to areas of low oxygen tensions such as type II muscle fibres (demonstrated in animal models [57, 58], but with less convincing data in humans [59–63]).

Current research has provided valuable insight into optimisation of NO_3_^−^ supplementation strategies (e.g. pharmacokinetics, dose-response and supplementation duration) [13, 64, 65] and mechanisms of action [51, 53, 57, 66]. Nevertheless, more research is needed to understand whether findings from typically small, well-controlled laboratory studies are likely to translate into clinically relevant improvements in population health. This article highlights key areas for further research that could help in this regard. Such research is warranted to help guide practitioners, influence policy, and form guidelines for the effective and safe consumption of inorganic NO_3_^−^.

## Main text

### Research focus 1: large-scale, longer duration trials

Although NO_3_^−^ consumption has been linked with a range of positive health outcomes, the majority of trials exploring the salutary effects of inorganic NO_3_^−^ have involved short-term supplementation regimens, typically a few days in duration. Only a handful of trials have explored the medium- to longer-term effects of NO_3_^−^ consumption (4 weeks to 6 months), usually focusing on BP or endothelial function as an outcome. Whilst not a universal finding [67, 68], beneficial effects of medium- to longer-term NO_3_^−^ supplementation protocols have been reported in some trials [69–71]. For example, Siervo et al. [69] found that 2 months supplementation with NO_3_^−^-rich beetroot juice (~ 6.5 mmol/d NO_3_^−^) decreased 24-h systolic and diastolic BP by − 10.8 and − 5.4 mmHg, respectively, in a Sub-Saharan African setting. Similar effects were also observed when NO_3_^−^ rich beetroot juice was co-ingested alongside folate. In another study, Mills and colleagues [70] showed that 6 months consumption of NO_3_^−^-rich beetroot juice (~ 11 mmol/d NO_3_^−^) decreased central systolic pressure by − 2.6 mmHg. Likewise, Kapil et al. [71] reported reductions in 24-h systolic and diastolic BP (7.7 and 5.2 mmHg, respectively) and improved endothelial function and arterial stiffness with 4 weeks NO_3_^−^-rich beetroot juice supplementation (6.4 mmol/d NO_3_^−^) with no change after placebo. Although focusing on different outcomes to the above trials, a study by Thompson et al. [72] also showed greater adaptations to sprint interval training in individuals consuming NO_3_^−^ rich beetroot juice (13 mmol/d) over a 4-week period, providing further evidence of a benefit of this supplement when given over prolonged periods.

By contrast, studies by Blekkenhorst [67] and Sundqvist [68] observed no effects of 4- and 5-week NO_3_^−^ interventions on BP. The lack of effect in these studies could be related to the relatively low NO_3_^−^ doses administered (2.4 and 4.8 mmol/d NO_3_^−^, respectively). Conversely, the source of NO_3_^−^ (vegetables or NO_3_^−^ pills rather than NO_3_^−^-rich beetroot juice) could be relevant in explaining the lack of effect in these studies, given different foods providing equivalent doses of NO_3_^−^ appear to have divergent effects on plasma NO_2_^−^ concentration and BP [16], which could be linked to the (poly) phenol and ascorbate content of these foods [73]. Indeed, in most studies to date NO_3_^−^ has been administered as beetroot juice, which is also rich in a constellation of different bioactive compounds, particularly (poly) phenols and the betalains [74]. Independent of NO_3_^−^, betalains have been shown to possess antioxidant [75], anti-inflammatory [76], and vasodilatory [77] properties, although studies in humans are still scarce. To isolate the effects NO_3_^−^ from other compounds in beetroot juice, researchers often compare the effects of a NO_3_^−^ rich beetroot juice to a taste-, smell- and appearance-matched NO_3_^−^ depleted juice. One limitation of this strategy is that it cannot account for any synergistic interactions between NO_3_^−^ and the other bioactive compounds that may augment the physiological effects of beetroot juice; in other words, we cannot be certain if the positive effects in these studies are simply due to NO_3_^−^ or its interactions with the other bioactive compounds present. Thus, studies chiefly aimed at untangling the mechanistic effects of NO_3_^−^ may prefer to administer NO_3_^−^ in the form of NO_3_^−^ salts instead of food-based supplements that contain other compounds. Overall, additional comparisons of NO_3_^−^ rich beetroot juice and sodium NO_2_^−^ or NO_3_^−^ supplements are required. When interpreting the findings of the studies discussed in this review, it is important the reader is aware that studies with NO_3_^−^ salts and NO_3_^−^ -rich beetroot juice do not contain the same compounds and therefore different effects are possible. Notwithstanding, as discussed in *Section 4* of this review, cross-talk between NO_3_^−^ and other dietary components or participant-level differences in the response to NO_3_^−^ could also account for the lack of effect of NO_3_^−^ in the studies of Blekkenhorst [67] and Sundqvist [68].

Based around the current evidence it is likely that, under the right circumstances (which remain to be fully elucidated), consumption of inorganic NO_3_^−^ could elicit longer-term health benefits. In order to fully appreciate the potential applications of NO_3_^−^ on population health, large-scale (e.g. n= > 1000), longer duration (e.g. 2–5 years) trials which focus not only on risk factors (e.g. BP, endothelial function, cognitive function), but also incidence of key non-communicable diseases (e.g. CVD, dementia) are warranted. Specific considerations for the design of such studies are provided in Table 1. Whilst likely to be logistically complex and require substantial financial backing from funders, this research could be justified by the promising evidence from short-term trials and the potential application of findings to ease the unsustainable societal and financial burden of conditions such as CVD (annual global costs ~$863 billion [78]) and dementia (annual global cost ~$1 trillion [79]). Prior to undertaking this research, it is essential to obtain more data on the feasibility and acceptability of different strategies to increase habitual NO_3_^−^intake by a sufficient quantity and for a sufficient period to obtain long lasting health benefits. This information is critical for the design of feasible longer-term trials and translation to the general population, and will be explored in more detail in the next section.

**Table 1:** Key considerations for future randomised controlled trials exploring the health effects of NO_3_^−^ ingestion

Consideration	Recommendation
Dose	Consumption of a NO_3_^−^ dose ≥8 mmol
NO_3_^−^ form	Provision of NO_3_^−^ salts or vegetables, with NO_3_^−^ content independently verified
Study duration	Longer duration (e.g., months-to-years) warranted
Participant cohort	‘At risk’ cohort studied (e.g., individuals with hypertension for studies exploring effects of NO_3_^−^ on cardiovascular disease risk)
Genetics/ microbiome	Consider recruitment of T allele carriers with G894T polymorphism in the eNOS gene
Microbiome	Consider recruitment of individuals with greater abundance of NO_3_^−^ reducing oral bacteria
Mouthwash	Avoidance of mouthwash prior to and during the study
Dietary controls	Avoidance of thiocyanate and sulphate rich foods in conjunction with NO_3_^−^
Other lifestyle factors	Avoidance of smoking
Outcomes	Inclusion of hard clinical endpoints (e.g., CVD or dementia incidence) to build upon promising findings on risk factors for these conditions

### Research focus 2: feasibility and acceptability of different strategies to facilitate prolonged, increased consumption of nitrate

To date, a limited number of studies have reported data on the feasibility and acceptability of beetroot juice as a vehicle for increasing habitual NO_3_^−^ intake. Mixed findings have been reported. For example, Ormesher and colleagues [80] gave 40 pregnant women 70 mL/d concentrated beetroot juice (~ 400 mg of NO_3_^−^) and, after 8 days of ingestion, 97% of participants indicated they would consume the supplement again, if they were experiencing benefits. However, only 62% of participants reported finding it easy to consume the beetroot juice and just over half of the participants rated the drink as palatable (54%). These findings suggest that longer-term consumption of beetroot juice may be difficult in this cohort, which could impede longer-term adherence. More recently, Kandhari et al. [81] evaluated the feasibility of a 60-day concentrated beetroot juice and folate intervention to treat hypertension in Sub-Saharan Africa. No serious adverse events were reported, and compliance was > 90%, suggesting beetroot juice was well accepted in this population. In addition, all participants rated the taste as “good” or “very good” and most participants (~ 87%) indicated a preference for beetroot juice over BP medication. The studies by Ormesher et al. [80] and Kandhari et al. [81] both administered the same brand of concentrated beetroot juice, such that the different findings cannot be attributed to a different type of supplement administered. Alternatively, it is possible that the different findings of Ormesher et al. [80], which was conducted in the UK, and Kandhari et al. [81], which was conducted in Tanzania, reflect cultural/ regional differences in food preference. However, it is noteworthy that participants in the Ormesher et al. [80] study were also pregnant, which may have further contributed towards the difference in palatability given pregnancy is known to influence taste [82].

In another study, Babateen et al. [83] examined the feasibility of different doses of concentrated beetroot juice in overweight and obese older adults over a 13-week period. Compliance was high, no adverse events were reported, and the attrition rate was 19%, which is similar or lower than the dropout rates reported in other human intervention trials [84, 85]. Collectively, these studies suggest that beetroot juice may represent an acceptable strategy to facilitate increased consumption of NO_3_^−^, at least in certain cohorts. However, future studies need to evaluate the feasibility and acceptability of beetroot juice consumption over longer periods (e.g., > 6 months) and in other populations.

Concentrated, commercially available beetroot juice shots have the advantage of being readily available (they are now sold in many major supermarket chains) and contain a standardised dose of NO_3_^−^ sufficient to influence myriad health outcomes. This form of beetroot juice has also been shown to be more effective at reducing BP (and presumably eliciting other physiological changes) than non-concentrated beetroot juice when the same dose is administered [86]. In addition, as mentioned in the previous section, beetroot juice also contains other bioactive compounds that may contribute to overall health. Nevertheless, as participants do not always enjoy the taste of beetroot juice and the relatively high cost of commercially available beetroot ‘shots’ (~£1–2 or $2–3 each) may be prohibitive to some users, it is essential for researchers to explore the feasibility and acceptability of other strategies to increase NO_3_^−^ consumption. This could include other NO_3_^−^-rich foods (e.g. lettuce, rhubarb, spinach, radish), gels, powders, crystals, capsules and non-beetroot drinks. To this end, both Blekkenhorst et al. [67] (> 98% compliance) and Sundqvist et al. [68] (> 97% compliance) demonstrated excellent compliance to 4 and 5 week interventions, respectively, with NO_3_^−^-rich vegetables, which were well tolerated with minimal side effects. Importantly, Sundqvist et al. [68] reported similar compliance between NO_3_^−^-rich vegetables and NO_3_^−^-containing pills (> 97% vs. > 98%). Nevertheless, neither Blekkenhorst et al. [67] nor Sundqvist et al. [68] reported beneficial physiological effects of their interventions, which could be related to the relatively modest NO_3_^−^ doses provided (~ 2.4 and 4.8 mmol/d respectively) or other methodological factors which were discussed in *Section 1* of this review. A comprehensive investigation of patient preferences and the real and perceived barriers of adopting a high-NO_3_^−^ diet or consuming NO_3_^−^-rich supplements warrants further investigation. In addition, studies need to determine the amount of NO_3_^−^-rich vegetables required to elicit beneficial physiological effects, whether this is achievable for different populations, and whether effects are superior to non-vegetable NO_3_^−^ sources. Finally, it is worth exploring whether there are regional and population preferences, as this knowledge could be used to develop more targeted NO_3_^−^ products.

### Research focus 3: nitrate intake and health outcomes in epidemiological studies

The role of dietary NO_3_^−^ for human health has gradually shifted over the last five decades. Indeed, this compound was initially considered as a risk factor for cancer, endocrine disorders and infant methaemoglobinaemia. However, the stigma attached to dietary NO_3_^−^ has gradually dwindled, and NO_3_^−^ is now viewed by many a potential health-promoting compound (see *Section 6* for further details). The initial results suggesting a harmful role of dietary NO_3_^−^ intake (from food) were mostly derived from animal models and weakly designed epidemiological studies which have had a prominent, almost demonizing, influence on defining the role of dietary NO_3_^−^ for human health [87]. These initial studies informed the still contentious WHO nutritional recommendations for dietary NO_3_^−^ intake in humans which was set at 3.7 mg/ kg body weight [88]. The perception of dietary NO_3_^−^ as a risk factor started to change with the discovery of the role of NO_3_^−^ as key substrate for the NO_3_^−^-NO_2_^−^-NO pathway and the evidence of a beneficial effect of NO_3_^−^ on health parameters such as BP.

After the study by Larsen et al. [10] in 2006, which first demonstrated a BP lowering effect of sodium NO_3_^−^, there was a rapid surge in research testing the effects of dietary NO_3_^−^ on health outcomes [89]. However, the research strategy in the last decade has almost taken an inverse approach to that typically adopted in nutritional science as the conduction of clinical trials have surpassed epidemiological investigations, which are generally considered as a first step to validate research hypotheses [90–92]. One of the primary reasons for the inverse trend is the lack of reliable and representative food databases of NO_3_^−^ content to support an accurate dietary assessment [93]. An additional limitation is the severe lack of validation studies testing the accuracy of dietary assessment methods against valid biomarkers of NO_3_^−^ intake (e.g. 24-h urinary NO_3_^−^ concentrations) [94]. This is compounded by the fact that the NO_3_^−^ content of vegetables will vary by farming method (whether NO_3_^−^ fertiliser is used or not), growing conditions, time of year the crop is harvested, and storage conditions [1], such that there is likely to be a degree of error in estimated NO_3_^−^ intake values [95]. Several research groups have developed independent databases by collecting data on NO_3_^−^ food content from published sources in an attempt to obtain valid estimates of NO_3_^−^ intake and evaluate associations with health outcomes [96, 97]. Although this is a step in the right direction, it remains difficult to accurately estimate long-term habitual dietary NO_3_^−^ intake for the reasons mentioned above. In addition, NO_3_^−^ concentrations measured in biological fluids have been used in some analysis as indirect markers of NO_3_^−^ intake [98]. Whether these objective markers of NO_3_^−^ intake show stronger links with health outcomes compared with subjective, self-reported NO_3_^−^ intake values, is the subject of ongoing research. A summary of the key non-cancer related epidemiological studies testing the association of inorganic NO_3_^−^ with health outcomes is provided in Table 2.

**Table 2:** Key epidemiological studies exploring associations between inorganic nitrate consumption and non-cancer related health outcomes

Author, year	Population Size	Study Design	Duration of Follow up (y)	Nitrate Assessment	Health Outcome	Key Findings
Bahadoran et al., [99]	4920	Prospective (Tehran Lipid and Glucose Study)	5.8	FFQ	Type 2 Diabetes (T2D)	No significant association between NO_3_^−^ intake and the risk of T2D in fully adjusted model
Kang et al. [100]	Nurses’ Health Study (63,893 women) Health Professionals Follow-up Study (41,094 men)	Prospective	~ 30 years for both	FFQ	Primary open-angle glaucoma (POAG)	Higher dietary NO_3_^−^ and green leafy vegetable intake was associated with a lower POAG risk, particularly POAG with early paracentral VF loss at diagnosis.
Mirmiran et al. [101]	1546	Prospective (Tehran Lipid and Glucose Study)	3	FFQ	Chronic Kidney Disease (CKD)	At baseline, higher intake of high-vegetable NO_3_^−^ intake was associated with a 48% higher chance of having CKD (OR 1.48, 95% CI 1.05–2.13). After 3 years of follow-up, there was no significant association with the occurrence of CKD
Blekkenhorst et al. [102]	1227	Prospective (Perth Longitudinal Study of Aging in Women)	15	FFQ	Atherosclerotic vascular disease (ASVD) mortality	A high vegetable NO_3_^−^ intake was associated with a lower risk of ASVD (HR: 0.79 95% CI: 0.68, 0.93, *P* = 0.004) and all-cause mortality (HR: 0.87 95% CI: 0.78, 0.97, *P* = 0.011)
Bondonno et al. [103]	1226	Prospective (Perth Longitudinal Study of Aging in Women)	14.5	FFQ	CCA-IMT, plaque severity and risk of an ischemic cerebrovascular disease event	Higher intake of vegetable NO_3_^−^ was associated with 17% lower risk of cerebrovascular disease events (*P* = 0.02) and lower CCA-IMT (*P* = 0.002).
Gumanova et al. [104]	1087	Cross-sectional (Stress Aging and Health Study)	–	Plasma NOx	Diabetes type II, hyperthyroidism, coronary heart disease, gout and thrombosis/stroke, osteoporosis, cancer	NOx over 44.7 μM were associated with increased prevalence of diabetes type II, hyperthyroidism, coronary heart disease, gout and thrombosis/stroke
Kuhnle et al. [105]	7598	Cross-sectional (EPIC Norfolk)	–	Drinking water NO_3_^−^ concentrations	Blood pressure (BP)	At low sulfate concentrations, NO_3_^−^ was inversely associated with BP (− 4 mmHg in top quintile) whereas this was reversed at higher concentrations (+ 3 mmHg in top quintile)
Maas et al. [106]	2855	Prospective (Framingham Offspring Study)	17.3	Plasma NO_3_^−^	All-cause mortality and incident CVD	Plasma NO_3_^−^ was weakly associated with an increased risk of death (HR, 1.16; 95%CI, 1.00–1.35 *P* = 0.057) but not with incident CVD
Smallwood et al. [107]	919	Cross-Sectional (InChianti)	–	24-h urinary NO_3_^−^	Blood pressure	Systolic blood pressure in the ≥2 mmol urinary NO_3_^−^ excretion group was 3.9 (CI: − 7.1 to − 0.7) mm Hg lower than in the comparison < 1 mmol excretion group.
Liu et al. [108]	2900	Prospective (Blue Mountains Eye Study)	15	FFQ	CVD mortality	In multivariable-adjusted analysis, participants in quartile 4 [> 137.8 mg/d; HR 0.63 (95% CI 0.41, 0.95)] of vegetable NO_3_^−^ intake had lower hazards for CVD mortality compared to participants in quartile 1 (< 69.5 mg/d)
Mendy et al. [109]	17,618	Prospective (NHANES)	4.3	Urinary NO_3_^−^ in spot urine samples	Hypertension and CVD prevalence and all-cause mortality	1-unit increase in log-transformed urinary NO_3_^−^ was associated with a > 30% decrease in the odds of hypertension (odds ratio, 0.67; 95% confidence interval [CI], 0.55–0.81), stroke (OR, 0.61, 95% CI, 0.43–0.87) and cardiovascular mortality (HR, 0.44; 95% CI, 0.26–0.73)
Jackson et al. [110]	5324	Prospective (Australian Longitudinal Study on Women’s Health)	15	FFQ	Incidence of self-reported CVD-related complications	Women reporting higher total dietary NO_3_^−^ intakes (Q4 > 78.2 mg/d) and vegetable NO_3_^−^ intakes (Q4 > 64.4 mg/d) were 25 and 27% reduced risk of developing CVD-related complications, respectively.
Jackson et al. [111]	Nurses’ Health Study and Health (62,535 women)	Prospective	26	FFQ	Coronary heart disease	Dietary NO_3_^−^ intake was not related to risk of CHD after adjustment for other lifestyle and non-vegetable dietary factors
Sim et al. [112]	1420	Cross-sectional (Perth Longitudinal Study of Aging in Women)	–	FFQ	Hand-grip strength and time up and go (TUG)	Higher NO_3_^−^ intake (31.2 mg/d) was associated with lower odds for weak grip strength (OR 0.84, 95% CI 0.74–0.95, *P* = 0.005) and slow TUG (OR 0.86, 95% CI 0.76–0.98, *P* = 0.021)
Riddell et al. [113]	2656	Prospective	1.5	Urinary NO_3_^−^ to creatinine ratio (uNCR)	Prediction of renal transplant rejection	Overall uNCR was highly variable with no diagnostic threshold for kidney transplant rejection
Wu et al. [114] 2020	14,894	Cross-sectional (NHANES)	–	Urinary NO_3_^−^ in spot urine samples	Congestive heart failure, coronary heart disease, angina pectoris, myocardial infarction	Significant association between urinary NO_3_^−^ and congestive heart failure (OR = 0.651, 95% CI 0.507–0.838, *P* < 0.001)
Pereira et al. [98]	1015	Cross-sectional (NHANES)	–	Urinary NO_3_^−^ in spot urine samples	Cognitive function	Urinary NO_3_^−^ concentrations were not associated with cognitive performance on any of the cognitive tests.

*EPIC* European Prospective Investigation of Cancer, *FFQ* Food Frequency Questionnaire, *CCA-IMT* Common Carotid Intimal Medial Thickness, *NO*_*3*_^*−*^ Nitrate, *NO*_*2*_^*−*^ nitrite, *NOx* Nitrate + Nitrite Concentration, *CVD* Cardiovascular Disease, *OR* Odds Ratio, *HR* Hazard Ratio, *NHANES* National Health and Nutrition Examination Survey, *uNCR* Urinary nitrate to creatinine ratio

The first studies to evaluate the association between dietary NO_3_^−^ intake and health outcomes were conducted in 2016 in Iran (two studies) [99, 101] and in the United States (one study) [100]. The former evaluated the association of vegetable NO_3_^−^ intake with risk of chronic kidney disease in the Tehran Lipid and Glucose Study and found a higher prevalence of chronic kidney disease (CKD) at baseline (cross-sectional analysis) in the high- NO_3_^−^ intake group whereas no significant association with CKD risk was observed after a 3-year follow up [101]. Using the same dataset, Bahadoran et al. [99] found that dietary NO_3_^−^ intake, overall and from animal sources, was not associated with prospective risk of diabetes. The US study was conducted in a very large sample (> 100,000 participants) and assessed dietary NO_3_^−^ intake in the Nurses’ Health Study and the Health Professionals Follow-up Study [100]. The results showed a significantly lower risk of primary open-angle glaucoma in participants with higher NO_3_^−^ intake [100]. However, a subsequent analysis conducted in the Nurses’ Health Study found a non-significant association between dietary NO_3_^−^ and prospective risk of coronary heart disease [111]. More recently, several cross-sectional and longitudinal studies have observed significant associations between high NO_3_^−^ intake or urinary NO_3_^−^ concentrations (as a proxy for NO_3_^−^ intake) with cardiovascular outcomes including lower BP [107], risk of hypertension [109], common carotid intimal medial thickness [103], congestive heart failure [114] and CVD mortality [109]. Conversely, higher plasma NO_3_^−^ concentrations in the Framingham Offspring Study [106] were associated with an increased risk of all-cause mortality, which may be explained by the rise in plasma NO_3_^−^ concentrations in participants with impaired kidney function included in the analysis and highlights the potential risk of reverse causality in these investigations. The improvements in physical performance and cognition observed in some of the NO_3_^−^ supplementation trials were also explored in two cross sectional studies [98, 112]. Improved hand-grip strength and timed up and go tests (a test of functional mobility) were observed in middle-aged and older Australian participants with a higher NO_3_^−^ intake [112] whereas NO_3_^−^ concentrations measured in spot urine samples were not associated with improved cognition in 1015 older Americans participants enrolled in the National Health and Nutrition Examination Survey [98]. The NIH workshop on dietary NO_3_^−^ held in 2016 [115] advocated for more epidemiological research to be conducted to better define the predictive role of dietary NO_3_^−^ consumption for the prevention as well as treatment of chronic diseases. The consensus statement also encouraged the development of detailed and country-specific NO_3_^−^ food composition tables for a more accurate assessment of the exposure to dietary NO_3_^−^ [115]. The current epidemiological evidence points towards a protective role of dietary NO_3_^−^ intake for cardiovascular events and mortality whereas the predictive role for cancer risk is still undefined as latest meta-analyses on the topic indicate a lack of association between dietary NO_3_^−^ consumption and cancer risk [116, 117]. There is still scarce or no data from prospective studies on the association of dietary NO_3_^−^ intake with other chronic conditions with established links with NO_3_^−^/NO_2_^−^ and NO pathways such as diabetes, hypertension, physical disability or dementia. Further epidemiological studies in this area are therefore warranted. Such research will complement the findings from RCTs, by providing information on the effectiveness of a NO_3_^−^ for disease reduction in real-world circumstances with greater sample sizes and longer follow up than is logistically feasible in most RCTs [90, 91].

### Research focus 4: inter-individual differences in the response to nitrate

At the individual participant level, several groups have suggested the existence of possible ‘responders’ and ‘non-responders’ to NO_3_^−^, irrespective of the vehicle used to provide this inorganic anion [64, 118, 119]. It is important to note that random within-subject variation could explain much of the variability in response to NO_3_^−^ supplementation between individuals [120, 121]. Similarly, issues may also arise when attempting to establish whether an individual is a dependable ‘responder’ or ‘non-responder’ on different occasions [122, 123]. Nevertheless, several factors have been identified which could explain genuine differences in the response to NO_3_^−^ between individuals. These include individual characteristics such as age [124, 125], health [126] and exercise training status [40, 49], sex [14], genetic factors [127], and differences in the oral microbiome (explored further in *Section 5* of this review). In addition, between-participant differences in potentially plastic lifestyle factors such as smoking status [128], use of mouthwash [129], and habitual diet [49, 130] might also impact an individual’s response to NO_3_^−^. We briefly review the impact of these variables on the effects of NO_3_^−^ below.

Individuals with lower aerobic fitness levels may respond more favourably to NO_3_^−^ supplementation [40, 131]. This theory stemmed from several studies reporting that while NO_3_^−^ supplementation from any source enhanced exercise performance in recreational level athletes (V̇O_2peak_ 40–60 ml/kg/min), such effects were less pronounced or non-existent in well-trained and elite endurance athletes (typically manifesting a V̇O_2max_ > 60 ml/kg/min) [132–135]. Porcelli et al. [40] provide the most convincing evidence to support this notion and demonstrated that, when all other methodological factors such as the exercise test and NO_3_^−^ dose are held content, individuals with a higher aerobic fitness status are less responsive to the ergogenic effects of NO_3_^−^. Indeed, those authors reported beneficial effects of sodium NO_3_^−^ on 3 km running performance in individuals with low (V̇O_2peak_: 28.2–44.1 ml/kg/min), and moderate (V̇O_2peak_: 45.5–57.1 ml/kg/min), but not high (V̇O_2peak_: 63.9–81.1 ml/kg/min) aerobic fitness levels. Several possible explanations have been put forth to try and explain why high fitness levels might render NO_3_^−^ supplementation less effective, and these are discussed in detail elsewhere [40, 131, 136]. One prominent explanation is that elite endurance athletes might produce more NO via the canonical NOS pathways and are therefore less reliant on NO_3_^−^ as a substrate for NO generation [132]. Furthermore, recent evidence indicates that NO_3_^−^ might elicit preferential effects on type II compared with type I muscle fibres [54, 57, 58]. Well-trained endurance athletes might therefore benefit less from NO_3_^−^ supplementation given a lower proportion of type II, and a higher proportion of type I, muscle fibres compared with recreationally active individuals [137, 138]. In contrast, some studies have shown a beneficial effect of NO_3_^−^ in well-trained athletes [42, 139–141]. Jonvik et al., (2015) suggested that methodological limitations of some studies could at least partly explain the null findings in some studies with elite athletes. Notably, there are far less studies assessing the effects of NO_3_^−^ supplementation, irrespective of vehicle, in well-trained athletes in comparison to healthy, physically active, individuals. This is likely because well-trained athletes are only a small fraction of the population, and are logistically harder to test and recruit to studies due to their desire to avoid potential training interruptions. Thus, more research is still required to ascertain the influence of aerobic fitness levels on the responsiveness to NO_3_^−^ supplementation.

Women are underrepresented in research into the health effects of dietary NO_3_^−^ [142]. Nevertheless, preliminary evidence suggests potentially differential effects of NO_3_^−^ (at least in regard to the effects of NO_3_^−^ on BP) between the sexes, which warrants further investigation. Women have been demonstrated to have greater oral NO_3_^−^ reducing capacity than men due to an oral microflora composition that is more conducive for NO_3_^−^ reduction to NO_2_^−^ [143]. Nevertheless, Kapil et al. [14] and Coles and Clifton [144] both demonstrated BP-lowering effects of NO_3_^−^ (potassium NO_3_^−^ and beetroot juice, respectively) in men with higher baseline BP and lower plasma NO_2_^−^ concentrations but not in women. Likewise, in a meta-analysis by Jackson et al. [17], BP reductions with NO_3_^−^ were greater in studies with more male participants. Those authors speculated that this could be related to a greater vascular production of NO in pre-menopausal women due to oestrogen-related release and activity of NO [145], diminishing the response to supplemental NO_3_^−^ in women compared with men.

Although studies remain scarce, there is some evidence that the heterogeneous responses to NO_3_^−^ supplementation are partly explained by polymorphisms in the eNOS gene. This was first explored by Hobbs et al., [127], who examined the effects of NO_3_^−^ supplementation on BP in patients with and without a specific polymorphism in the eNOS gene (G894T), which has been suggested to inhibit NO production from eNOS [127]. Although findings are equivocal [146], the G894T polymorphism, alongside being a T allele carrier, has been associated with cardiovascular disease [147–149], of which a key risk factor is diminished NO bioavailability [150, 151]. Intriguingly, despite the small sample size (*n* = 14), Hobbs et al., [127] found that NO_3_^−^ supplementation (beetroot bread) only reduced BP in patients who were both T allele carriers and had the G894T polymorphism in the eNOS gene. A more recent study examined the influence of the G894T polymorphism and NO_3_^−^ therapy on mortality in chronic heart failure patients [146]. Somewhat at odds with the findings of Hobbs et al., [127], Azzam et al. [146] found that NO_3_^−^ therapy (source not specified) increased the risk of mortality in patients with the G894T polymorphism, and to a greater extent in G allele carriers, suggesting that NO_3_^−^ therapy might increase mortality in advanced heart failure. However, as this study was observational, cause-effect relationships cannot be established. Moreover, the findings are at contrast to the beneficial effects of NO_3_^−^ shown in most [152–155], but not all [156, 157], short term intervention trials which show that NO_3_^−^ improves cardiac function and/or exercise capacity in heart failure patients. Clearly, more studies with larger cohorts are required to determine the extent to which genetic variation influences the responsiveness to NO_3_^−^ supplementation, but the findings from these two studies raise the possibility that that genetic factors could contribute towards the inter-individual variability reported by many studies.

Smoking has been shown to increase plasma and salivary concentrations of thiocyanate [158], a compound which competitively inhibits uptake of NO_3_^−^ into the salivary glands [159], potentially reducing the amount of ‘substrate’ available to the oral bacteria for reduction into NO_2_^−^. Consequently, it is possible that smokers will experience compromised NO_3_^−^ metabolism and thus a diminished physiological response to NO_3_^−^ supplementation versus non-smokers. Indeed, Bailey et al. [128] demonstrated a smaller increase in salivary NO_3_^−^, plasma NO_3_^−^ and NO_2_^−^ concentration, and an attenuated BP response, following a NO_3_^−^ bolus (beetroot juice) in smokers compared to non-smoking controls.

It is possible that supplemental NO_3_^−^ is ineffective at eliciting meaningful physiological changes in individuals habitually consuming a high NO_3_^−^ diet. Nevertheless, as population intake of NO_3_^−^ is typically low — Babateen et al. [93] reported a median intake of 108 mg/d in healthy individuals. With very few individuals regularly consuming NO_3_^−^ levels to match those provided through supplementation [160], high habitual NO_3_^−^ intake is unlikely to explain a lack of response to NO_3_^−^ supplementation in most ‘non-responders’. Alternatively, there is compelling evidence to suggest that consumption of other dietary compounds alongside NO_3_^−^ may have the capacity to influence response to this compound, such that an individual’s background diet could determine (at least transiently) their status as a NO_3_^−^ ‘responder’ or ‘non-responder’. For example, consumption of glucosinolate-rich vegetables, such as those from the *Brassica* family like broccoli, cauliflower, and cabbage, proximal to consumption of NO_3_^−^-rich vegetables was shown to blunt the BP lowering response of the latter [130]. Interestingly, this appears to be related to a similar mechanism to which smoking attenuates the effect of NO_3_^−^. Specifically, during processes that result in plant cell membrane damage such as mastication, glucosinolates are exposed to the enzyme myrosinase, which catalyses the hydrolysis of glucosinolates into thiocyanate [161]. Although consumption of thiocyanate-rich vegetables leads to lower salivary and plasma thiocyanate concentrations compared with smoking, Dewhurst-Trigg et al. [130] showed that the BP-lowering effect of a NO_3_^−^-rich smoothie was attenuated by the presence of thiocyanate rich vegetables. In that study, thiocyanate did not seem to interfere with NO_3_^−^ transport into the mouth (as evident by similar salivary NO_3_^−^ concentrations when NO_3_^−^ was consumed alongside vegetables that were both high and low in thiocyanate), suggesting that thiocyanate may influence other aspects of NO_3_^−^ metabolism. Specifically, co-ingestion of thiocyanate synthesising vegetables and NO_3_^−^-rich vegetables lowered salivary NO_2_^−^ concentration compared to ingestion of NO_3_^−^-rich vegetables alone. This suggests that some *Brassica* vegetables might transiently alter the oral microbiome, consistent with the antimicrobial effects of thiocyanate derivatives in the oral cavity [162].

A study by Hughan et al. [163] found that the co-ingestion of sodium NO_3_^−^ alongside conjugated linoleic acid, an unsaturated fatty acid particularly abundant in dairy and meat products, attenuated the rise in plasma NO_3_^−^ and NO_2_^−^ concentrations and supressed the BP-lowering and platelet-inhibiting effects that were apparent when supplements were administered in isolation. Mechanistically, co-consumption of conjugated linoleic acid altered the metabolic fate of ingested NO_3_^−^ leading to the formation of conjugated linoleic acid nitration products, which do not appear to have the same vasodilatory and platelet inhibiting properties as NO_2_^−^ and NO. Likewise, Bailey et al. [164] found that the ingestion of iodide, which is fortified in many foods [165] and known to compete for salivary NO_3_^−^ uptake [159], lowered salivary NO_3_^−^ concentration when co ingested with NO_3_^−^ rich beetroot juice. However, the increase in salivary and plasma NO_2_^−^ concentration, alongside the lowering of BP, were similar compared with NO_3_^−^ alone. Finally, a possible interaction between dietary NO_3_^−^ and sulphate was identified by Kuhnle et al. [105] who indicated that when estimated sulphate intake was low, higher dietary NO_3_^−^ intake was associated with lower BP. Conversely, when sulphate intake was high, this association was reversed, such that greater NO_3_^−^ intake was actually associated with higher BP. The mechanistic basis through which sulphate could modulate the BP lowering effects of dietary NO_3_^−^ is presently unknown.

Collectively, the evidence presented above indicates that the response to NO_3_^−^ is unlikely to be uniform between individuals, and could also potentially differ within individuals based around malleable lifestyle factors such as habitual diet. Better understanding the factors that influence responsiveness to NO_3_^−^ is crucial to maximise the efficacy of NO_3_^−^-based interventions and will facilitate the development of targeted interventions for individuals most likely to benefit from consumption of this compound. Given many of the factors which appear to moderate the effectiveness of NO_3_^−^ impact the oral conversion of this compound into NO_2_^−^, future research could also explore the potential physiological effects of direct NO_2_^−^ administration (for a recent example, see [166]), which does not require processing in the mouth and could theoretically elicit more consistent responses between individuals. Nevertheless, caution should be taken to ensure such a strategy does not increase formation of potentially carcenogenic nitrosamines [167].

### Research focus 5: oral microbiota and oral health

Once in the oral cavity, NO_3_^−^ is reduced to NO_2_^−^ during the anaerobic respiration of facultative and obligate bacteria which are particularly abundant on the dorsal surface of the tongue [168]. The oral microbiome collectively comprises over 700 individual species or phylotypes of bacteria that are organised in a series of complex interdependent communities [169]. To date, 14 species of bacteria have been identified as NO_3_^−^ reducers, the majority of which are from the genera *Veillonella*, *Prevotella, Neisseria,* and *Haemophilus* [170]*.* A greater relative abundance of these bacteria on the tongue has been shown to augment the rate and magnitude of salivary NO_2_^−^ production following the ingestion of NO_3_^−^ rich beetroot juice [171]*.* Conversely, disruption of the oral microbiome by antibacterial mouthwash causes a transient loss of viable NO_3_^−^-reducing bacteria [172] and severely blunts the generation of NO_2_^−^ in the saliva [173]. Strong antibacterial mouthwash has also been shown to increase BP, likely due to suppression of NO production from the NO_3_^−^-NO_2_^−^-NO pathway [174–176]. These data confirm the essential role of the oral bacteria in NO homeostasis and support the hypothesis that oral and systemic health are inextricably linked [177].

The mouth is continually exposed to the external environment and is regularly subjected to brushing, flossing, and nutrient intake, all of which may influence the physiological conditions inside the oral cavity and alter the composition of the bacterial milieu [178]. Ageing is known to cause a reduction in salivary flow rate [179] and has been reported to alter the composition of the oral microbiome in some [180, 181] but not all [182] studies. Other factors may also be expected to influence the abundance and activity of oral bacteria, including exercise, diet, oral and systemic diseases, haemodialysis [183] and peritoneal dialysis [184] and medication (particularly antibiotics). In particular, the ingestion of NO_3_^−^-rich beetroot juice has been shown to increase salivary pH and cause meaningful alterations to the oral microbiome in favour of oral health [182, 185]. Given the multitude of potential modifiers, it is perhaps unsurprising that there is profound between-individual variation in the abundance of NO_3_^−^-reducing bacteria [121]. Of note, these authors also reported significant within-individual week-to-week variability in the abundance of these bacteria and the magnitude by which plasma NO_2_^−^ increased following the ingestion of NO_3_^−^-rich beetroot juice. This was despite participants standardising their diet, physical activity, use of mouthwash, teeth brushing, and tongue cleaning between visits. The unpredictability in how different individuals respond to NO_3_^−^ supplementation and how the same individual responds across repeated visits poses a particular challenge for researchers who wish to explore the therapeutic effects of this dietary intervention.

While recent advancements in genomic sequencing techniques have greatly enhanced our understanding of human bacterial interactions in the context of NO homeostasis, several important questions remain unanswered. To date, the majority of the research exploring links between the oral microbiome and health outcomes has only reported the relative abundance of phyla, genera, or species. Although this quantifies the proportional makeup of the community structure it does not reveal the metabolic activities of individual bacterial species [186] which may vary depending on substrate availability, metabolite expression from neighbouring microbes and host cells, and the impact of environmental conditions [187]. Future research should deploy meta-transcriptomic analysis to determine how factors such as diet, medication, physical activity, ageing, and disease influence NO_2_^−^ and NO_3_^−^ reductase gene expression of the oral bacteria. Furthermore, data from epidemiological studies and short-term intervention trials seem to support the notion that increasing habitual dietary intake of NO_3_^−^ can improve markers of oral health and reduce the incidence of caries [185, 188, 189]. It remains to be established whether dietary NO_3_^−^ supplementation may also be an effective treatment method for those already suffering from oral diseases such as chronic periodontitis.

### Research focus 6: risks versus rewards

NO_3_^−^ is increasingly recognised as a beneficial ion that protects against chronic disease, yet, as noted in *Section 3* of this review, historically, it was considered a food contaminant with adverse health effects, particularly increased risk of certain cancers and methaemoglobinaemia [1, 88]. While the aforementioned WHO ADI for NO_3_^−^ of 3.7 mg/kg of body mass remain in place today, the discovery of multiple positive health effects of NO_3_^−^ have prompted a re-examination of these claims.

In 2004 the WHO reaffirmed their restrictions on NO_3_^−^ intake yet, in 2008, a panel of experts from the European Food Safety Authority, concluded that the epidemiological evidence did not support an association between NO_3_^−^ and cancer risk [190]. Similarly, in 2010, the International Agency for Research on Cancer confirmed that there was inadequate evidence to suggest NO_3_^−^ from food or water was carcinogenic in humans [191]. Evidence that NO_3_^−^ might cause infant methemoglobinemia, which was first mooted in the 1940s [192], has also been questioned. Indeed, an investigation conducted on behalf of the WHO in 2004 found no exposure-response relationship between dietary NO_3_^−^ and methemoglobinemia in infants [193]. It is also worth noting that although some studies report mild adverse symptoms with high NO_3_^−^ intake such as nausea and sickness, to the authors knowledge, no serious adverse events have ever been reported in clinical trials administering NO_3_^−^ [1, 93].

Notwithstanding, the available evidence does not rule out the possibility that prolonged consumption of NO_3_^−^ above the ADI could harm health. Currently, at least with short to medium term intakes, research suggests that doses exceeding the ADI are needed to optimise vascular health or exercise performance [17, 48]. Because most human trials have only examined the acute health effects (< 4 weeks) of increased NO_3_^−^ intake, the long-term safety of consuming NO_3_^−^ in amounts that exceed the ADI is not well understood. At present, epidemiological studies provide the strongest evidence that prolonged, high intakes of NO_3_^−^ are safe. Indeed, these indicate that rather than being harmful, dietary NO_3_^−^ intake is inversely associated with cardiovascular disease risk [102, 194] and certain cancers [117]. Furthermore, diets and dietary patterns high in fruits and vegetables are linked to greater longevity [195, 196], protection against type 2 diabetes [197] and chronic obstructive pulmonary disease [198], and improved cardiovascular [92, 199, 200] and cognitive health [201, 202]. This suggests that higher intake of dietary NO_3_^−^, at least through plants, is more likely to be associated with health benefits than adverse effects.

Some animal studies have explored the longer-term effects of high dietary NO_3_^−^ intake on health. In a study in rats, 10 weeks of a low sodium NO_3_^−^ dose (0.1 mmol/kg/d), which the authors suggest is equivalent to amounts achievable in the human diet, reduced BP, whereas a much higher dose (1 mmol/kg/d), elevated BP [203]. Interestingly, this study found that the high NO_3_^−^ dose down-regulated eNOS activity, not only suggesting a crosstalk between the canonical and NO_3_^−^- NO_2_^−^-NO pathway, but also that any vascular benefits afforded by NO_3_^−^ supplementation could wane over time. Nonetheless, these findings were not supported by a more recent animal study from the same group. Hezel and colleagues [204] fed mice the human equivalent of 350 mg/d or 26 mg/d of sodium NO_3_^−^. After 17 months, mice consuming the high NO_3_^−^ diet did not have elevated BP or any other adverse health effects, despite the fact the dose exceeded the WHO recommended ADI for an adult under ~ 95 kg. On the contrary, the high NO_3_^−^ diet decreased plasma insulin and modulated inflammation, findings consistent with the metabolic benefits observed in acute human studies [205]. These effects need to be verified in humans but support the notion that prolonged increases in NO_3_^−^ intake are not harmful to health.

It is important to note that any carcinogenic risk attributed to NO_3_^−^ intake could be mitigated by the intake of antioxidants such as vitamin C or (poly) phenols, which are present in most fruits and vegetables. Studies have shown that vitamin C and E are effective inhibitors of nitrosamine formation [167]. In addition, (poly) phenols, which are abundant in commonly consumed NO_3_^−^ sources such as spinach and beetroot [206], can also abrogate nitrosamine formation [73]. Thus, increasing NO_3_^−^ intake through a greater vegetable intake may significantly lessen the risk of any NO_3_^−^ induced nitrosamine formation. This could partly explain why diets high in vegetables are associated with a reduced and not heightened risk of cancer.

Health concerns have also been raised over the high oxalate content of NO_3_^−^-rich vegetables [207, 208]. Oxalates are present in several foods, but particularly high in spinach, beetroot, and rhubarb [208, 209]. Intake of these foods increases urinary oxalate excretion, a risk factor for renal stone formation [209–211], thus, it is currently recommended that foods rich in dietary oxalates are consumed in moderation [208, 210]. However, the link between dietary oxalates and kidney stone formation remains equivocal. Although consuming oxalate rich foods increases oxalate excretion, a large prospective study (> 190,000 participants) found only modest non-significant associations between dietary oxalate intake and kidney stone risk, concluding that dietary oxalate intake is not a major risk factor for the formation of kidney stones in younger or older adults [212]. Furthermore, the Dietary Approaches to Stop Hypertension (DASH) diet, which is high in oxalates and NO_3_^−^-rich vegetables [1], was recently shown to increase urinary oxalate excretion but reduce the risk of kidney stone formation in ~ 260 patients [213]. The authors attributed these findings to the high calcium and magnesium content of the diet limiting oxalate absorption. This is supported by previous research showing that oxalates from beetroot have low bioavailability (< 1%), owing to their high calcium content [209]. While more prospective human trials are needed, evidence that oxalate rich vegetables increase the risk of kidney stone formation is limited.

To summarise, claims that dietary NO_3_^−^ promotes cancer or methemoglobinemia, or that dietary oxalates cause kidney stones are weak and unsubstantiated. Rather, there is compelling evidence that dietary NO_3_^−^ has salutary health effects and warrants consideration as a long-term therapeutic treatment strategy to manage vascular and metabolic health. Notwithstanding, longer-term studies in humans are lacking and thus it cannot be ruled out that a prolonged increase in NO_3_^−^ intake, above the WHO recommended ADI, may have adverse effects for some individuals. Thus, it is incumbent that researchers examine the long-term safety of increasing dietary NO_3_^−^ consumption in a range of contexts and populations. This research will be vital for convincing the public and regulators that NO_3_^−^ consumption is safe and that current recommendations to limit dietary NO_3_^−^ intake should be re-considered.

## Conclusions

This article has briefly outlined the current state of knowledge around the potential health effects of dietary inorganic NO_3_^−^. Six key areas worthy of future research were identified to enhance understanding of the potential role of NO_3_^−^ in improving population health. As such, it is hoped that this article will help direct researchers to further explore the role of NO_3_^−^ as a potential health-promoting dietary component.

## Data Availability

Not applicable.

## Abbreviations

ADI: Acceptable daily intake
BP: Blood pressure
CVD: Cardiovascular disease
CKD: Chronic kidney disease
DASH: Dietary approach to stop hypertension
eNOS: Endothelial nitric oxide synthase
NIH: National Institutes of Health
NO_3_^−^: Nitrate
NO: Nitric oxide
NO_2_^−^: Nitrite
V̇O_2peak_: Peak oxygen uptake
WHO: World Health Organisation
